# Supplementary material for: Modeling glioblastoma heterogeneity as a dynamic network of cell states
Source: Mol Syst Biol. 2021 Sep 16;17(9):e10105. doi: 10.15252/msb.202010105 (PMC8444284; doi:10.15252/msb.202010105)
Supplement: Supplementary file 5 — Source Data for Figure 3 [file MSB-17-e10105-s001.zip › Figure3A_sourcedata/GSEA_3065/nosigdb_state5.GseaPreranked.1622718435914/LARSSON_STATE_1.html]

Details for gene set LARSSON\_STATE\_1[GSEA]

|  || Dataset | state5 |
| Phenotype | NoPhenotypeAvailable |
| Upregulated in class | na\_neg |
| GeneSet | LARSSON\_STATE\_1 |
| Enrichment Score (ES) | -0.7993353 |
| Normalized Enrichment Score (NES) | -2.7595947 |
| Nominal p-value | 0.0 |
| FDR q-value | 0.0 |
| FWER p-Value | 0.0 |
Table: GSEA Results Summary

  

Fig 1: Enrichment plot: LARSSON\_STATE\_1      
 Profile of the Running ES Score & Positions of GeneSet Members on the Rank Ordered List

  

| PROBE | GENE SYMBOL | GENE\_TITLE | RANK IN GENE LIST | RANK METRIC SCORE | RUNNING ES | CORE ENRICHMENT || 1 | SCG2 |  |  | 166 | 0.470 | -0.0093 | No |
| 2 | CTSV |  |  | 193 | 0.443 | -0.0034 | No |
| 3 | POSTN |  |  | 316 | 0.375 | -0.0097 | No |
| 4 | CTSC |  |  | 347 | 0.362 | -0.0059 | No |
| 5 | SPP1 |  |  | 724 | 0.256 | -0.0431 | No |
| 6 | DCBLD2 |  |  | 863 | 0.230 | -0.0541 | No |
| 7 | DKK1 |  |  | 1388 | 0.163 | -0.1098 | No |
| 8 | SYNJ2 |  |  | 1418 | 0.160 | -0.1099 | No |
| 9 | TMEFF2 |  |  | 1546 | 0.147 | -0.1213 | No |
| 10 | IGFBP3 |  |  | 2037 | 0.109 | -0.1742 | No |
| 11 | IGFBP5 |  |  | 3230 | 0.040 | -0.3076 | No |
| 12 | ANK3 |  |  | 3818 | 0.015 | -0.3734 | No |
| 13 | SLC14A1 |  |  | 3984 | 0.008 | -0.3918 | No |
| 14 | PYGB |  |  | 5874 | -0.069 | -0.6030 | No |
| 15 | USP53 |  |  | 6269 | -0.089 | -0.6456 | No |
| 16 | PTHLH |  |  | 6317 | -0.091 | -0.6490 | No |
| 17 | NETO2 |  |  | 6583 | -0.107 | -0.6767 | No |
| 18 | PMAIP1 |  |  | 6872 | -0.128 | -0.7066 | No |
| 19 | PLP2 |  |  | 6975 | -0.135 | -0.7154 | No |
| 20 | CCK |  |  | 7003 | -0.137 | -0.7157 | No |
| 21 | VIPR1 |  |  | 7051 | -0.142 | -0.7182 | No |
| 22 | CDC42EP3 |  |  | 7562 | -0.199 | -0.7716 | No |
| 23 | CAV1 |  |  | 7575 | -0.200 | -0.7690 | No |
| 24 | TXNRD1 |  |  | 7767 | -0.228 | -0.7860 | No |
| 25 | PMEPA1 |  |  | 7830 | -0.241 | -0.7882 | No |
| 26 | UPP1 |  |  | 7849 | -0.243 | -0.7853 | No |
| 27 | FBXO32 |  |  | 7866 | -0.247 | -0.7822 | No |
| 28 | TWIST1 |  |  | 8019 | -0.279 | -0.7938 | Yes |
| 29 | MAP3K7CL |  |  | 8063 | -0.288 | -0.7929 | Yes |
| 30 | CAPN2 |  |  | 8080 | -0.293 | -0.7889 | Yes |
| 31 | TKT |  |  | 8122 | -0.301 | -0.7875 | Yes |
| 32 | TWF2 |  |  | 8217 | -0.330 | -0.7915 | Yes |
| 33 | LDHA |  |  | 8263 | -0.343 | -0.7898 | Yes |
| 34 | STK17A |  |  | 8267 | -0.345 | -0.7832 | Yes |
| 35 | CTNNBIP1 |  |  | 8270 | -0.345 | -0.7766 | Yes |
| 36 | CCND1 |  |  | 8346 | -0.368 | -0.7777 | Yes |
| 37 | GCHFR |  |  | 8348 | -0.369 | -0.7705 | Yes |
| 38 | TUBB4B |  |  | 8356 | -0.371 | -0.7639 | Yes |
| 39 | EIF6 |  |  | 8362 | -0.374 | -0.7570 | Yes |
| 40 | HOXB2 |  |  | 8363 | -0.375 | -0.7495 | Yes |
| 41 | CLIC1 |  |  | 8368 | -0.378 | -0.7425 | Yes |
| 42 | PLIN3 |  |  | 8403 | -0.388 | -0.7386 | Yes |
| 43 | ARL4C |  |  | 8410 | -0.390 | -0.7315 | Yes |
| 44 | PTTG1 |  |  | 8446 | -0.406 | -0.7274 | Yes |
| 45 | LMNA |  |  | 8478 | -0.423 | -0.7225 | Yes |
| 46 | TMEM158 |  |  | 8499 | -0.434 | -0.7161 | Yes |
| 47 | MMP24OS |  |  | 8539 | -0.452 | -0.7115 | Yes |
| 48 | C12orf75 |  |  | 8546 | -0.456 | -0.7031 | Yes |
| 49 | WDR1 |  |  | 8554 | -0.463 | -0.6947 | Yes |
| 50 | NQO1 |  |  | 8588 | -0.479 | -0.6889 | Yes |
| 51 | ANXA1 |  |  | 8595 | -0.484 | -0.6799 | Yes |
| 52 | COTL1 |  |  | 8612 | -0.501 | -0.6717 | Yes |
| 53 | PDLIM4 |  |  | 8630 | -0.512 | -0.6635 | Yes |
| 54 | CCDC85B |  |  | 8643 | -0.521 | -0.6545 | Yes |
| 55 | CAST |  |  | 8645 | -0.523 | -0.6442 | Yes |
| 56 | TUBA1C |  |  | 8648 | -0.524 | -0.6340 | Yes |
| 57 | CAVIN1 |  |  | 8657 | -0.531 | -0.6243 | Yes |
| 58 | ARPC5 |  |  | 8669 | -0.540 | -0.6149 | Yes |
| 59 | PRDX6 |  |  | 8673 | -0.540 | -0.6045 | Yes |
| 60 | HMGA1 |  |  | 8675 | -0.543 | -0.5938 | Yes |
| 61 | DRAP1 |  |  | 8691 | -0.552 | -0.5845 | Yes |
| 62 | DPYSL3 |  |  | 8692 | -0.554 | -0.5735 | Yes |
| 63 | S100A16 |  |  | 8739 | -0.596 | -0.5668 | Yes |
| 64 | S100A11 |  |  | 8767 | -0.626 | -0.5574 | Yes |
| 65 | AP1S2 |  |  | 8782 | -0.636 | -0.5463 | Yes |
| 66 | SPATS2L |  |  | 8793 | -0.645 | -0.5346 | Yes |
| 67 | PHLDA1 |  |  | 8805 | -0.655 | -0.5228 | Yes |
| 68 | S100A13 |  |  | 8820 | -0.670 | -0.5111 | Yes |
| 69 | S100A6 |  |  | 8827 | -0.681 | -0.4982 | Yes |
| 70 | SMS |  |  | 8834 | -0.690 | -0.4852 | Yes |
| 71 | ODC1 |  |  | 8839 | -0.697 | -0.4717 | Yes |
| 72 | PRELID1 |  |  | 8847 | -0.705 | -0.4585 | Yes |
| 73 | GADD45GIP1 |  |  | 8849 | -0.709 | -0.4445 | Yes |
| 74 | ACTG1 |  |  | 8859 | -0.718 | -0.4313 | Yes |
| 75 | ANXA2 |  |  | 8868 | -0.729 | -0.4177 | Yes |
| 76 | ARPC1A |  |  | 8879 | -0.744 | -0.4040 | Yes |
| 77 | SRM |  |  | 8880 | -0.746 | -0.3892 | Yes |
| 78 | DSTN |  |  | 8885 | -0.752 | -0.3747 | Yes |
| 79 | GAP43 |  |  | 8890 | -0.759 | -0.3600 | Yes |
| 80 | PPP1R14B |  |  | 8900 | -0.771 | -0.3457 | Yes |
| 81 | UBE2S |  |  | 8901 | -0.772 | -0.3303 | Yes |
| 82 | TPM4 |  |  | 8905 | -0.778 | -0.3152 | Yes |
| 83 | ACTB |  |  | 8912 | -0.793 | -0.3001 | Yes |
| 84 | OAZ1 |  |  | 8922 | -0.824 | -0.2848 | Yes |
| 85 | MYL6 |  |  | 8938 | -0.851 | -0.2695 | Yes |
| 86 | TPI1 |  |  | 8939 | -0.852 | -0.2526 | Yes |
| 87 | TXN |  |  | 8952 | -0.885 | -0.2363 | Yes |
| 88 | CSRP2 |  |  | 8954 | -0.891 | -0.2187 | Yes |
| 89 | SH3BGRL3 |  |  | 8955 | -0.895 | -0.2009 | Yes |
| 90 | PKM |  |  | 8959 | -0.915 | -0.1831 | Yes |
| 91 | HINT1 |  |  | 8961 | -0.923 | -0.1648 | Yes |
| 92 | S100A10 |  |  | 8965 | -0.932 | -0.1466 | Yes |
| 93 | RPL8 |  |  | 8971 | -0.992 | -0.1275 | Yes |
| 94 | RPSA |  |  | 8973 | -1.005 | -0.1076 | Yes |
| 95 | RPS12 |  |  | 8975 | -1.034 | -0.0872 | Yes |
| 96 | PFN1 |  |  | 8977 | -1.045 | -0.0665 | Yes |
| 97 | TMSB10 |  |  | 8979 | -1.090 | -0.0449 | Yes |
| 98 | LGALS1 |  |  | 8982 | -1.135 | -0.0226 | Yes |
| 99 | TMSB4X |  |  | 8983 | -1.142 | 0.0001 | Yes |
Table: GSEA details [plain text format]

  

Fig 2: LARSSON\_STATE\_1: Random ES distribution      
 Gene set null distribution of ES for **LARSSON\_STATE\_1**
